# Activation of Mu or Delta Opioid Receptors in the Lumbosacral Spinal Cord Is Essential for Ejaculatory Reflexes in Male Rats

**DOI:** 10.1371/journal.pone.0121130

**Published:** 2015-03-31

**Authors:** Natalie Kozyrev, Lique M. Coolen

**Affiliations:** 1 Department of Anatomy & Cell Biology, the University of Western Ontario, London, Ontario, Canada; 2 Department of Physiology, University of Michigan, Ann Arbor, Michigan, United States of America; 3 Department of Neurobiology & Anatomical Sciences, University of Mississippi Medical Center, Jackson, Mississippi, United States of America; 4 Department of Physiology & Biophysics, University of Mississippi Medical Center, Jackson, Mississippi, United States of America; University of New South Wales, AUSTRALIA

## Abstract

Ejaculation is controlled by a spinal ejaculation generator located in the lumbosacral spinal cord, consisting in male rats of lumbar spinothalamic (LSt) cells and their inter-spinal projections to autonomic and motor centers. LSt cells co-express several neuropeptides, including gastrin releasing peptide (GRP) and enkephalin. We previously demonstrated in rats that GRP regulates ejaculation by acting within the lumbosacral spinal cord. In the present study, the hypothesis was tested that enkephalin controls ejaculation by acting on mu (MOR) or delta opioid receptors (DOR) in LSt target areas. Adult male rats were anesthetized and spinalized and received intrathecal infusions of vehicle, MOR antagonist CTOP (0.4 or 4 nmol), DOR antagonist (TIPP (0.4, 4 or 40 nmol), MOR agonist DAMGO (0.1 or 10 nmol), or DOR agonist deltorphin II (1.3 or 13 nmol). Ejaculatory reflexes were triggered by stimulation of the dorsal penile nerve (DPN) and seminal vesicle pressure and rhythmic contractions of the bulbocavernosus muscle were analyzed. Intrathecal infusion of MOR or DOR antagonists effectively blocked ejaculatory reflexes induced by DPN stimulation. Intrathecal infusion of DAMGO, but not deltorphin II triggered ejaculation in absence of DPN stimulation. Both MOR and DOR agonists facilitated ejaculatory reflexes induced by subthreshold DPN stimulation in all animals. Overall, these results support the hypothesis that enkephalin plays a critical role in the control of ejaculation in male rats. Activation of either MOR or DOR in LSt target areas is required for ejaculation, while MOR activation is sufficient to trigger ejaculation in the absence of sensory stimulation.

## Introduction

Ejaculation is a complex physiological phenomenon that is highly rewarding and culminates in the ejection of seminal contents from the urethral meatus [1–3]. Ejaculation involves two phases, emission and expulsion. During the emission phase, seminal fluids are secreted from the accessory sex glands, including the prostate, seminal vesicles and vas deferens and the bladder neck and external urethral sphincter shut to avert retrograde ejaculation. During the expulsion phase, rhythmic contractions of the striated perineal muscles, and the bulbocavernosus muscle in particular, forcefully expel semen from the urethral meatus [4–7]. Ejaculation is a reflex controlled by a spinal ejaculation generator in the lumbosacral spinal cord [2,8]. The spinal ejaculation generator is thought to control ejaculation by closely coordinating sympathetic, parasympathetic, and motor components to initiate emission and expulsion [3,9–13]. In addition, it is postulated that the spinal ejaculation generator triggers ejaculation by integrating sensory inputs conveyed by the dorsal penile nerve (DPN), the sensory branch of the pudendal nerve, during copulation with autonomic and motor outflow [14–16]. The spinal ejaculation generator consists of a set of interneurons in lamina 10 and the medial portion of lamina 7 of lumbar segments 3 and 4 (L3-4) that are integral in the control of ejaculation. These lumbar interneurons are referred to as lumbar spinothalamic (LSt) cells due to their anatomical position in the lumbar spinal cord and projections to the subparafascicularparvocellular nucleus of the thalamus [17–20]. One proposed role of LSt cells is to transform sensory signals associated with the summation of sexual activity into motor or secretory outputs [17]. LSt cells display neural activation specifically with ejaculation but not with other components of sexual behavior following copulation [20] or following electrical stimulation of the dorsal penile nerve (DPN) in male rats [14]. Markers of neural activation in LSt cells with ejaculation include cFos [20] and phosphorylated extracellular signal-regulated kinases 1 and 2 (pERK) [14] and ejaculation is triggered through activation of the MAP kinase pathway [14] following mating in intact animals or DPN stimulation in anesthetized and spinalized male rats. Furthermore, LSt cells have axonal projections to LSt target regions including preganglionic sympathetic, parasympathetic and motor neurons in the thoracolumbar and lumbosacral spinal cord [12,17, 21–24].

LSt cells express several neuropeptides including galanin, cholecystokinin, gastrin releasing peptide (GRP), and enkephalin [17–20]. Thus, we hypothesize that these neuropeptides act in the LSt target areas to trigger or modulate ejaculatory reflexes. Indeed, studies from our laboratory revealed a critical role for GRP in the control of ejaculation in male rats [25]. Intrathecal infusions of GRP antagonist RC-3095 completely abolished ejaculatory reflexes while intrathecal infusion of GRP agonist GRP^2029^ triggered ejaculatory reflexes in 43–66% of animals and facilitated ejaculatory reflexes in response to subthreshold frequencies (5–10 Hz) of DPN stimulation in 100% of male rats [25]. In the current study we tested if the opioid peptide enkephalin is critical for regulation of ejaculation. Previously, it was reported that intravenous injections of the opioid agonist morphine blocked ejaculatory reflexes in male rats and that this effect was reversed by systemic pretreatment with the opioid receptor antagonist naloxone [26]. Furthermore, intravenous naloxone induced the expression of the genital ejaculatory motor pattern [26]. Thus, it was concluded that endogenous opioids exert inhibitory effects on ejaculatory function. However, the non-specific nature of the opioid agonist and antagonist used in this previous study as well as the intravenous mode of drug delivery constitute significant confounds in the interpretation of the results. In addition, it has been proposed that morphine inhibits ejaculation via action on peripheral tissues [27]. Enkephalin, which is expressed in LSt cells and axons [28], is an endogenous ligand for both mu (MOR) and delta (DOR) opioid receptors [29], and can thus trigger ejaculation by acting on either or both MOR and/or DOR in LSt target regions. Hence, the purpose of the present study was to investigate the individual contributions of MOR and DOR on ejaculatory reflexes induced by electrical stimulation of the DPN in anesthetized and acutely spinalized male rats, a physiologically relevant paradigm to study ejaculatory reflexes [30]. DPN stimulation reliably induces ejaculation in all mammals, including rodents [8], primates [31], and men [32]. Acute transection of the spinal cord allows for examination of ejaculatory reflexes in the absence of supraspinal influences [30]. Moreover, LSt cells and their intraspinal projections to autonomic and motor areas are critically involved in the ejaculatory reflexes induced by DPN stimulation in male rats, further validating the use of the DPN stimulation paradigm to study ejaculatory reflexes in rats [16]. First, we tested whether enkephalin is required for ejaculatory reflexes in male rats using intrathecal delivery of MOR and DOR antagonists during simultaneous recordings of seminal vesicle (SV) pressure, a marker of emission, and rhythmic contractions of the bulbocavernosus muscle (BCM), a marker of expulsion, in anesthetized and acutely spinalized male rats. Next, it was tested whether enkephalin is sufficient to trigger ejaculation in male rats in the absence of DPN stimulation or following subthreshold levels of DPN stimulation (5–10 Hz) that do not trigger ejaculatory reflexes in male rats in control conditions [27] and intrathecal infusions of specific MOR and DOR agonists in the lumbosacral spinal cord during recordings of SV pressure and BCM contractions.

## Materials and Methods

### Animals

Adult male Sprague Dawley rats (225–250 grams) were acquired from Charles River (Wilmington, MA, USA) and pair-housed in standard housing cages on a 12-hour light/dark cycle with lights off at 9pm.Food and water were available ad libitum. All procedures were approved by the University Committee on Use and Care of Animals at the University of Michigan and the University of Western Ontario and conformed to the guidelines outlined by the National Institutes of Health.

### Pharmacological Experiments

#### Surgical procedures

Procedures were similar to our previous studies [14–16,25]. Male rats were anesthetized with urethane (1.5 g/kg, i.p.) and a laminectomy was performed between the sixth and the eighth thoracic spinal segments. A complete transection of the spinal cord was performed at the seventh thoracic spinal segment. Subsequently, animals were prepared for electromyographic (EMG) recordings of bulbocavernosus muscle (BCM) bursting and seminal vesicle pressure (SVP); indicators of expulsion and emission components of ejaculation respectively [8,30,33]. The BCM and the dorsal penile nerve (DPN) were surgically exposed and the surrounding connective tissue was removed. Silver recording electrodes, connected to the PowerLab/7SP Data Acquisition System (AD Instruments, Inc., Colorado Springs, CO, USA) were placed bilaterally into the BCM and a ground electrode was inserted into the muscle of the right thigh. In a subset of animals, the right seminal vesicle was exposed by coeliotomy and a pressure catheter (AD Instruments Inc., model number: SPR-671(1.4 F, Single, Straight, 15 cm, Ny) connected to a catheter interface cable (AD Instruments Inc., model number: AEC-IOD) and attached to a Bridge AMP (AD Instruments Inc.), was gently guided into the lumen of the SV and fixed in place. Finally, for stimulation of the DPN, a bipolar stimulating electrode connected to the SD9 Square Pulse Stimulator (Grass Technologies, West Warwick, RI, USA) was arranged directly above the DPN. 2–4 Hours prior to any of the experiments, the DPN was stimulated (4 V at 60 Hz square wave pulses of 1 ms duration, for total of 10 seconds) in order to verify the completeness of the spinal cord transection. These established stimulation parameters consistently trigger rhythmic bursting of the BCM, indicative of the expulsion phase of ejaculation, in all animals [14–16, 25].

#### Intrathecal infusion of opioid receptor antagonists

Pharmacological experiments began at least two hours following transection of the spinal cord in order to allow the potential acute effects of spinal cord transection to diminish. A minor incision was performed in the dura mater caudally in the site of the laminectomy and a polyethylene catheter (Caly-Adams PE-10, Parsippany, NJ, USA) was guided into the subarachnoid space until the open end reached the mid-lumbar levels, such that saline or drug infused through the catheter would bathe the entire lumbosacral spinal cord. First, 10 μL of saline was delivered (at 10 μl/minute rate) and BCM EMG activity was recorded for 25 minutes. Next, the DPN was stimulated at 30 Hz and 60 Hz (4 V, square wave pulses of 1 ms duration, for a total of 10 seconds) in a counterbalanced manner, with 5-minutes between the stimulations (testing trial 1; control trial). BCM EMG and SVP were recorded. An hour later, the procedure was performed again in the same animals, which were now administered a 10 μL infusion of opioid receptor antagonists, after which the DPN was stimulated at 30 Hz and 60 Hz in a counterbalanced manner, and BCM EMG activity and SVP were recorded (testing trial 2; drug trial). We previously established that repeated stimulations and infusions of saline did not affect BCM EMG bursting in response to or without DPN stimulation [25], therefore all animals received drug treatment in testing trial 2 (drug trial). In experiment 1, one of two doses of the mu opioid receptor antagonist H-D-Phe-Cys-Tyr-D-Trp-Orn-Thr-Pen-Thr-NH_2_ (CTOP; Bachem Americas, Inc. Torrance, CA, USA) 0.4nmol (N = 7) or 4nmol (N = 8) were infused and BCM EMG (but not SVP) was recorded. In experiment 2, the most effective dose of CTOP was administered, i.e. 4 nmol, and BCM EMG and SVP were recorded simultaneously (N = 6). In experiment 3, one of three doses of the delta opioid receptor antagonist H-Tyr-Tic-Phe-Phe-OH (TIPP), [Bachem Americas, Inc. Torrance, CA, USA], 0.4 nmol (N = 6), 4 nmol (N = 7), or 40 nmol (N = 6) were infused and BCM EMG and SVP were recorded.

#### Intrathecal infusion of opioid receptor agonists

Next, it was tested whether activation of opioid receptors is sufficient to trigger ejaculation in male rats in the absence of DPN stimulation or following subthreshold levels of DPN stimulation (5–10 Hz) that do not trigger ejaculatory reflexes in male rats in control conditions [25]. In addition, the effects of opioid receptor agonists were tested on the DPN stimulation parameters that do reliably trigger ejaculatory reflexes (30 and 60 Hz).Four separate groups of male rats were anesthetized, spinalized, and prepared as described above. In testing trial 1 (control trial), all male rats received 10 μL intrathecal infusions of 0.9% saline (N = 30) and BCM EMG and SVP were recorded for 10 minutes after infusion. Next, the DPN was stimulated at 5 HZ, 10 HZ, 30 HZ and 60 HZ in a randomized and counterbalanced order, and BCM EMG and SVP were recorded for the duration of 90 seconds following stimulation (testing trial 1; control trial). We previously determined that repeated stimulation using these parameters did not affect BCM EMG [25]. One hour following the final DPN stimulation, the procedure was repeated in the same groups of animals, which now received 10 μL intrathecal infusions of one of two doses of mu opioid receptor agonist (D-Ala2, N-Me-Phe4, glycinol5)—Enkephalin [DAMGO 0.1 nmol, N = 7; 10 nmol, N = 8; (Bachem Americas, Inc. Torrance, CA, USA)] or one of two doses of the delta opioid receptor agonist [DAla2] Deltorphin II, 1.3 nmol, N = 8; 13 nmol, N = 8; [American Peptide Co, Sunnyvale, CA, USA] and BCM EMG and SVP were recorded for 10 minutes after infusion (testing trial 2; drug trial). Finally, the DPN was stimulated at 5, 10, 30, and 60 HZ in a counterbalanced manner as described above and previously (testing trial 2; drug trial [25]).

#### Analysis: BCM EMG and SVP increases

BCM EMG and SVP recordings were analyzed using methods we previously described [25]. Briefly, data were analyzed for the 25 or 10 minutes following the infusion of the antagonist or agonist, respectively. In addition, EMG and SVP were analyzed for 90 seconds following each DPN stimulation, which is the time span of a characteristic DPN-stimulation induced ejaculatory reflex in control male rats [14–16,25]. The numbers of events, bursts, and SVP increases were analyzed (see [25]) using LabChart 7.35 (AD Instruments Inc.). An event is defined as an increase in EMG above baseline; a burst is a cluster of EMG events (10 or more events) without return to baseline; and an increase in SVP is counted each time it occurs concurrently with BCM EMG activity. These definitions were previously determined [25].

For the antagonist experiments, the effects of antagonist treatments on the numbers of events, bursts and SVP increases were statistically compared using Two-Way Repeated Anova (factors: Testing trial and Drug dose) and Holm-Sidak post hoc tests, separately for each 30 and 60 Hz stimulation, both within animals (within each drug dose group, but between testing trial 1 (control trial) and testing trial 2 (drug trial)) and between groups (between different drug dose groups, but within testing trial 1 or within testing trial 2). For the agonist experiments, numbers of events, bursts and SVP increases were compared separately for each stimulation setting (following infusion without stimulation, 5, 10, 30, 60 Hz), using a Two-Way Repeated Anova (factors: Testing trial and Drug Dose) and Holm-Sidak post hoc tests, both within animals (between testing trial 1 and testing trial 2) and between treatment groups (between different dose groups within testing trial 1 or within testing trial 2). A 95% confidence level was used for all tests.

### Immunohistochemistry Experiment

To verify that mu opioid receptor antagonist CTOP and delta opioid receptor antagonist TIPP did not block ejaculation by preventing the activation of LSt cells, expression of phosphorylation of ERK (pERK) in LSt cells was examined following stimulation of the DPN [14,25]. Furthermore, previous experiments have shown that pERK is not expressed in LSt cells under baseline conditions and that spinalization and the surgical exposure of the DPN and BCM in the absence of electrical stimulation of the DPN do not induce pERK expression in LSt cells [14]. Therefore, pERK expression in LSt cells is specifically induced by ejaculatory behavior or DPN stimulation causing ejaculatory reflexes and negative control groups were not included in this experiment. Male rats (N = 9) were anesthetized and spinalized as described above. Two hours after spinalization, the DPN and BCM were exposed and vehicle (saline, N = 4) or drug (CTOP 4nmol; N = 3, TIPP 40 nmol; N = 2, Bachem Americas, Inc. Torrance, CA, USA) were infused in a volume of 10 μL intrathecally as described above. Following a 25-minute infusion, the DPN was stimulated at 30 Hz and BCM EMG activity and SVP were recorded for 90 seconds. Five minutes following DPN stimulation, animals were perfused transcardially with 5 mL 0.9% saline solution and 500 mL of 4% paraformaldehyde in 0.1 M PB. Spinal cords were removed and postfixed for 1hour in the same fixative and then transferred into a cryoprotective solution (20% sucrose in 0.1 PB with 0.01% sodium azide) until further processing for immunohistochemical visualization of pERK and galanin (marker for LSt cells, as it is expressed exclusively in LSt cell bodies [17,20]. The stimulation parameters and time of perfusion were previously shown to be optimal for detection of pERK expression in LSt cells [14,25].

#### Immunohistochemistry: Galanin/pERK dual fluorescence

Spinal cords were cut using a freezing microtome (Thermo Fisher Scientific, Walldorf, Germany) into 12 parallel series of 35 μm coronal sections in cryoprotectant solution (30% sucrose, 30% ethylene glycol in 0.1 M PB with 0.01% sodium azide) and stored at -20°C until further processing. Free floating sections of the lower thoracic, lumbar, and sacral spinal levels were thoroughly rinsed in 0.1 M saline buffered sodium phosphate (PBS) between incubations and blocked with 1% H2O2 for 10 minutes before incubation. All antibody incubations were performed in incubation solution containing 0.1% bovine serum albumin and 0.4% Triton X-100 (BP151-500,Thermo Fisher Scientific Inc, Pittsburgh, PA, USA) in PBS at room temperature with gentle agitation. Spinal cord sections were incubated overnight with rabbit anti-pERK (1:1,000; Cell Signaling #9101; Phospho-p44/42 MAPK (Erk1/2) (Thr202/Tyr204) and with biotinylated goat anti-rabbit (1:500; 1 hour; Vector Laboratories, Burlingame, CA, USA), avidin horseradish peroxidase complex (ABC-elite, 1:1,000 in PBS; 1 hour; Vector Laboratories), biotinylated tyramine/tissue sample amplification (TSA; 1:250 in PBS containing 1 uL/mL of 3% H2O2; 10 minutes; NEL700/700A; PerkinElmer LifeSciences, Boston, MA, USA), and Alexa 488-conjugated streptavidin (1:100 in PBS; 30 minutes (Jackson ImmunoResearch Laboratories, WestGrove, PA, USA). Next, sections were incubated with rabbit anti-galanin (1:3,000; overnight, T-4334; Bachem,Torrance, CA, USA) and Alexa 555-conjugated goat anti-rabbit (1:100 in PBS; 30 minutes; Jackson Immuno-Research Laboratories) and mounted on plus charged slides, cover-slipped with gelvatol and stored in the dark at 4°C. For all immunohistochemistry procedures, the omission of primary antibodies resulted in a complete loss of signal at specific wavelengths and all primary antibodies have been previously validated [14,25].

#### Analysis: pERK Expression in LSt cells

pERK expression in LSt cells was analyzed on a DM5000B Leica fluorescent microscope (Leica Microsystems, Wetzlar, Germany). Specifically, all lumbar spinal cord cells expressing galanin-ir were analyzed for expression of pERK, as cytoplasmic galanin-ir is exclusively expressed in LSt cells [20]. Data are expressed as the mean percentages of LSt cells that display pERK for each animal. The group means were calculated and compared between animals that received an infusion of CTOP (4 nmol) or TIPP (40 nmol) and saline-treated controls, using One Way ANOVA with 95% confidence levels.

## Results

### Mu Opioid Receptor Antagonist CTOP Suppressed Ejaculatory Reflexes

#### Experiment 1

Mu opioid receptor antagonist CTOP significantly decreased DPN stimulation-induced ejaculatory reflexes at both 30 and 60 Hz stimulation frequencies as reflected in significantly reduced numbers of BCM events and bursts.

**Fig 1:**
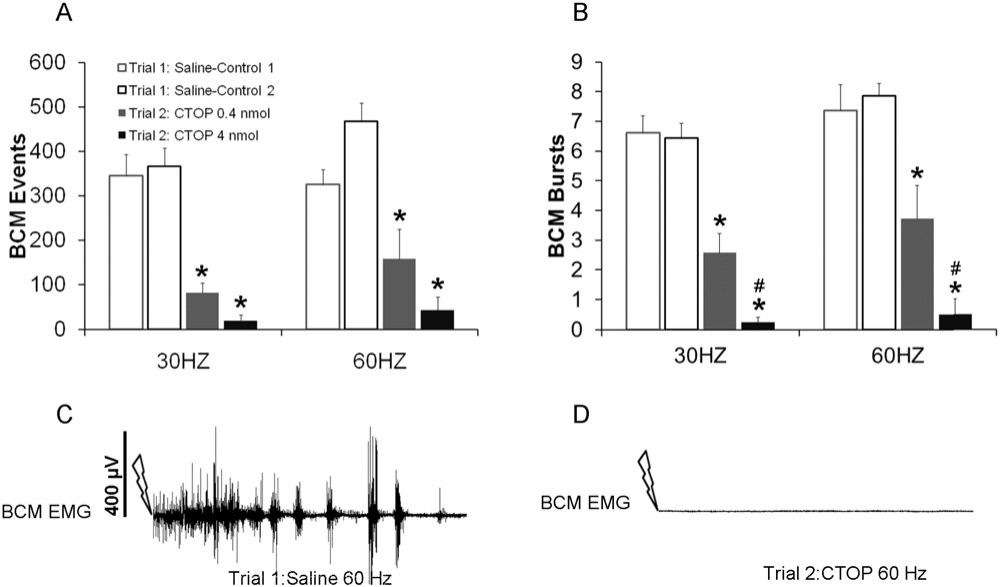
Quantitative analyses of BCM events (A) and bursts (B) in response to 30 and 60 Hz DPN stimulation following infusions of saline in trial 1 (Saline-Controls 1 and 2; white bars) or one of two doses of CTOP (0.4 or 4 nmol) in trial 2 (drug trial; filled bars). Trials 1 and 2 were conducted in the same animals, hence Saline-Control 1 are data for animals subsequently receiving CTOP 0.4 nmol in trial 2, and Saline-Control 2 are data for animals receiving CTOP 4 nmol. Data are presented as Mean ± SEM. * denotes significant differences from trial 1 within the same treatment group, while # indicates significant differences between treatment groups within the same testing trial. Representative BCM EMG traces (90 seconds duration) following 60 Hz DPN stimulation (arrow) after an intrathecal infusion of saline (**C**: control trial) and CTOP (**D**: same animal as in C).

Two-Way ANOVA revealed main effects of testing trial (control trial 1 versus drug trial 2) on the numbers of BCM events for both the 30 Hz (F(1, 29) = 88.436; *P* < 0.001; Fig. 1A) and 60 Hz stimulation frequencies (F(1, 29) = 62.013; *P* < 0.001; Fig. 1A). Post hoc analyses revealed that animals treated with either dose of CTOP during the testing trial 2, i.e. drug trial, had significantly decreased BCM events in response to 30 or 60 Hz DPN stimulation compared to their DPN stimulation induced BCM events following saline treatment in the first control testing trial (trial 1; 60 Hz: *P* <0.001 (0.4 nmol); *P* = 0.009 (4 nmol); 30 Hz: *P* = <0.001(0.4 nmol); *P* <0.001 (4 nmol); Fig. 1A). There were no significant effect of drug dosage and no differences between the two groups treated with the lower and higher doses of CTOP for each stimulation frequency.

Similarly, there were main effects of testing trial on the numbers of BCM bursts for both 30 Hz (F(1, 29) = 108.732; *P* < 0.001; Fig. 1B) and 60 Hz (F(1, 29) = 123.431; *P* < 0.001; Fig. 1B) stimulation frequencies. In addition, there were significant interactions between testing trial and drug dosage for both stimulation frequencies (30Hz: F(1.29) = 11.83; *P =* 0.023; 60 Hz: F(1,29) = 23.87; *P =* 0.016). CTOP during trial 2 (drug trial) significantly reduced BCM bursts compared to trial 1 (control trial) with saline treatment (30 Hz: *P* < 0.001 [0.4nmol], 30 Hz: *P* < 0.001 [4 nmol], 60 Hz: *P* < 0.001 [0.4 nmol], 60 Hz: *P* < 0.001 [4nmol]; Fig. 1B, C, D). There was an effect of dose on the numbers of BCM bursts in testing trial 2: drug trial. Specifically, there were significantly fewer BCM bursts following the higher dose compared to the lower dose of CTOP (30 Hz: *P* = 0.003; Fig. 1B, 60 Hz: *P* = 0.006; Fig. 1B). Finally, during the control testing trial (trial 1), there were no significant differences between groups in the numbers of BCM events or bursts.

#### Experiment 2

**Fig 2:**
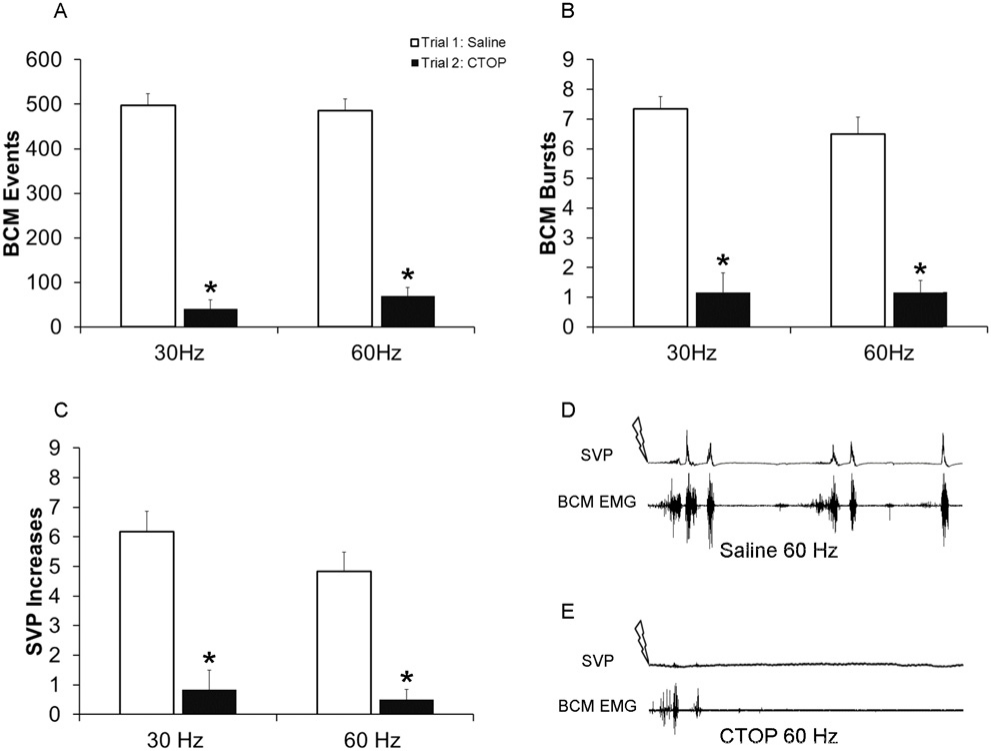
Quantitative analyses of BCM events (A) bursts (B) and SVP increases (C) in response to 30 and 60 Hz DPN stimulation following infusions of saline in trial 1 (control trial; white bars) or CTOP (4 nmol) in trial 2 (drug trial; filled bars). Data are presented as Mean ± SEM. * denotes significant differences from trial 1 (control trial). BCM EMG and concurrent SVP traces of 90 seconds duration following 60 Hz DPN stimulation (arrow) after an intrathecal infusion of saline (**D** control trial) and CTOP (**E** same animal as in D).

In a separate group of males, experiment 1 was repeated and expanded to show that CTOP (4 nmol) severely impaired the emission component of ejaculation as indicated by significantly decreased numbers of SVP increases (Fig. 2), in addition to impairment of the expulsion component evidenced by reduced BCM events and bursts (Fig. 2). There were main effects of testing trial on the numbers of SVP increases (F(1, 23) = 120.143; *P <*0.001; Fig. 2C, D, E), as well as BCM events (F(1, 23) = 513.688; *P* < 0.001; Fig. 2A, D, E), and BCM bursts (F(1, 23) = 118.433; *P* < 0.001; Fig. 2B, D, E). Post hoc analyses revealed that CTOP (4 nmol) in trial 2 (drug trial), significantly reduced numbers of SVP increases, BCM events and bursts compared to control trial 1, for both 30 Hz (events: *P <*0.001; bursts: *P <* 0.001; SVP: *P <* 0.001; Fig. 2A-E) and 60 Hz (events: *P <*0.001; bursts: *P <* 0.001; SVP: *P <* 0.001; Fig. 2A-E). Overall, these results confirm the hypothesis that intrathecal CTOP suppresses both the emission and expulsion components of ejaculation and that activation of mu opioid receptors in the lumbosacral spinal cord is required for ejaculatory reflexes in male rats.

### Delta Opioid Receptor Antagonist TIPP Suppressed Ejaculatory Reflexes

Similar to the effects of the mu opioid antagonist, inthrathecal infusion of delta opioid receptor antagonist TIPP significantly decreased DPN stimulation-induced ejaculatory reflexes at both 30 and 60 Hz stimulation frequencies as reflected in the reduced numbers of BCM events, BCM bursts, and SVP increases.

**Fig 3:**
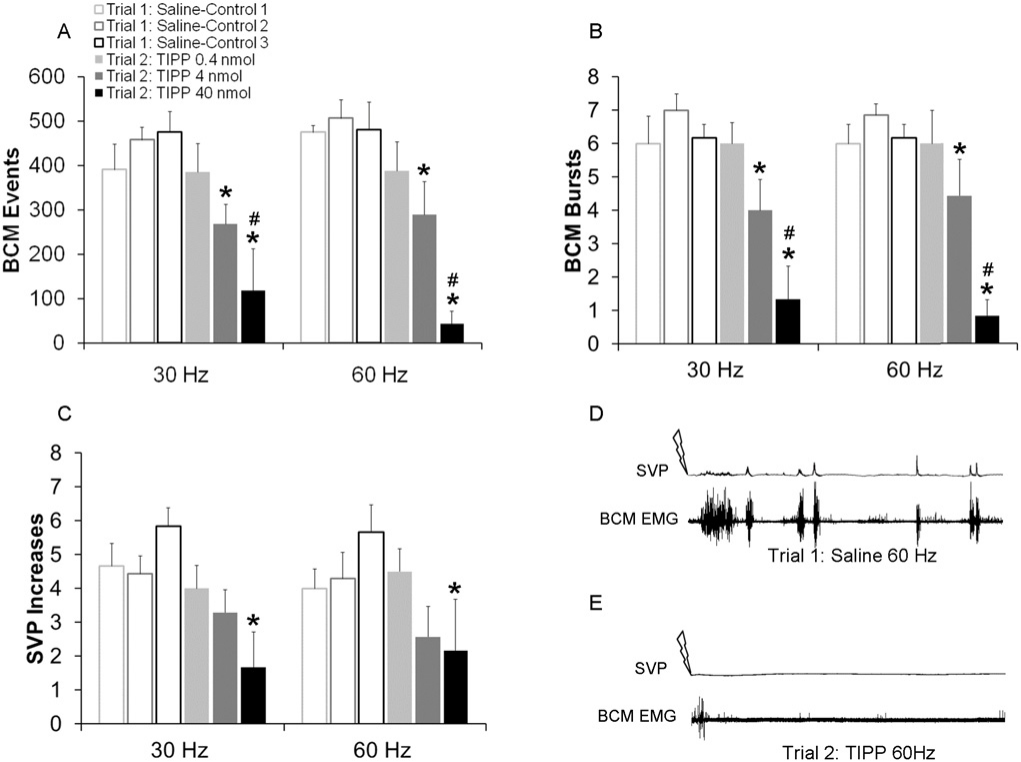
Quantitative analyses of BCM events (A) bursts (B) and SVP increases (C) in response to 30 and 60 Hz DPN stimulation following infusions of saline in trial 1 (Saline-Controls 1–3; white bars) or one of three doses of TIPP (0.4, 4 or 40 nmol) in trial 2 (drug trial; filled bars). Trials 1 and 2 were conducted in the same animals, hence Saline-Control 1 are data for animals subsequently receiving TIPP 0.4 nmol in trial 2; Saline-Control 2 are data for animals receiving TIPP 4 nmol in trial 2; and Saline-Control 3 are data used for animals receiving TIPP 40 nmol in trial 2. Data are presented as Mean ± SEM. * denotes significant differences from trial 1 within the same treatment group, while # indicates significant differences between treatment groups within the same testing trial. BCM EMG traces of 90 seconds duration following 60 Hz DPN stimulation (arrow) after an intrathecal infusion of saline (**D**: control trial) and TIPP (**E**: same animal as in C).

There were main effects of testing trial on the numbers of BCM events for both 30 Hz (F(1,37) = 20.131; *P* < 0.001; Fig. 3A) and 60 Hz stimulation frequencies (F(1, 37) = 26.666; *P* < 0.001; Fig. 3A), main effect of drug dosage for 60 Hz (F2,37) = 6.18; P = 0.01) and significant interactions between testing trial and drug dosage for both 30 and 60 Hz (30 Hz: (F(2,37) = 5.861; *P* = 0.012; 60 Hz ((F(2,37) = 4.349; *P* = 0.031). Animals treated with the middle (4 nmol) and higher (40 nmol) but not the lower (0.4 nmol) dose of TIPP during trial 2 (drug trial) had significantly decreased BCM events in response to 30 Hz (*P* = 0.012: 4 nmol; *P* = <0.001: 40 nmol; Fig. 3A) and 60 Hz (60 Hz: *P* = 0.014, 4 nmol; *P* <0.001, 40 nmol; Fig. 3A) stimulation frequencies compared to saline treatment in trial 1 (control trial). In addition, there was an effect of dosage, as animals treated with the highest dose of TIPP (40 nmol) in the second trial (drug trial) displayed significantly fewer BCM events compared to those treated with the lower dose of TIPP (0.4 nmol) for both the 30 Hz (*P* = 0.004, 40 nmol; Fig. 3A) and 60 Hz (*P* < 0.001, 40 nmol; Fig. 3A) stimulation frequencies and compared to animals treated with the middle dose of TIPP (4 nmol) in response to the 60 Hz (*P* = 0.003, 4 nmol; Fig. 3A) stimulation (with a trend for the 30 Hz (*P* = 0.075, 4 nmol; Fig. 3A)).

Similarly, numbers of BCM bursts were reduced for both 30 Hz (F(1, 37) = 23.209; *P* < 0.001; Fig. 3B) and 60 Hz (F(1, 37) = 21.298; *P* < 0.001; Fig. 3B) stimulation frequencies. Males treated with the middle (4 nmol) and the higher (40 nmol) but not the lower (0.4 nmol) dose of TIPP during trial 2 (drug trial) had significantly fewer BCM bursts compared to trial 1 (control trial) with saline treatment (30 Hz: *P* = 0.004 [4 nmol]; Fig. 3B, 30 Hz: *P* < 0.001 [40 nmol]; Fig. 3B; 60 Hz: *P* = 0.018 [4 nmol]; Fig. 3B, 60 Hz: *P* < 0.001 [40 nmol]; Fig. 3B, D, E).

Finally, TIPP reduced SVP increases and there were main effects of testing trial for both 30 Hz (F(1, 37) = 19.041; *P* < 0.001; Fig. 3C) and 60 Hz (F(1, 37) = 9.059; *P* = 0.008; Fig. 3C) stimulation frequencies. Males treated with the highest (40 nmol) but not the middle (4 nmol) or lower (0.4 nmol) doses of TIPP during trial 2 (drug trial) had significantly fewer SVP increases compared to trial 1 (control trial) with saline treatment (30 Hz: *P* < 0.001 [40 nmol], 60 Hz: *P* = 0.002 [40nmol]; Fig. 3C, D, E).

There were no significant differences for any of the ejaculation parameters between the groups of animals during trial 1 (control trial) when the groups received saline, indicating that group differences in trial 2 were caused by drug treatment.

### CTOP and TIPP Did Not Affect DPN Stimulation-Induced pERK in LSt Cells

**Fig 4:**
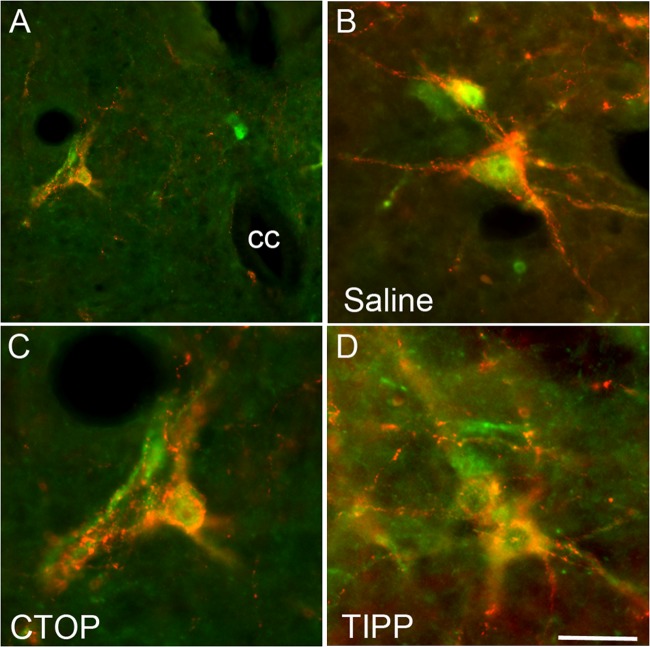
Opioid receptor antagonist infusions did not prevent the activation of pERK in LSt cells by DPN stimulation in male rats. Images demonstrate pERK expression (green) induced by DPN stimulation in LSt cells (visualized by galanin immunoreactivity; red) and the general location of the LSt cells, lateral to the central canal (cc) in laminae X and VII (A; in CTOP-treated male). Images show representative pERK-positive LSt cells following infusions of saline (B), the mu opioid receptor antagonist CTOP (C), or the delta opioid receptor antagonist TIPP (D). The pattern of pERK labeling is characteristic of all CTOP, TIPP, or saline-treated animals. Scale bar indicates 50 (A) or 15 (B-D) μm.

In order to test that mu opioid receptor antagonist CTOP and delta opioid receptor antagonist TIPP suppressed BCM bursting and SV pressure via actions in LSt target areas rather than by preventing activation of LSt cells, DPN stimulation-induced activation of LSt cells was examined. Previous experiments have shown that pERK expression in LSt cells is specifically induced by DPN stimulation or by ejaculatory behavior, and is not present in baseline conditions [14]. DPN stimulation induced pERK in control saline-treated males, in 95.4 ± 2.7% of LSt cells (Fig. 4), as we reported previously [14,25]. Neither CTOP or TIPP infusions prior to DPN stimulation affected LSt activation and percentages of LSt cells expressing DPN stimulation-induced pERK in CTOP- (96.5 ± 3.5% of LSt cells; Fig. 4) or TIPP-treated (100 ± 0% of LSt cells; Fig. 4) males did not significantly differ from saline-treated controls.

### Mu Opioid Receptor Agonist DAMGO Triggered and Facilitated Ejaculatory Reflex

**Fig 5:**
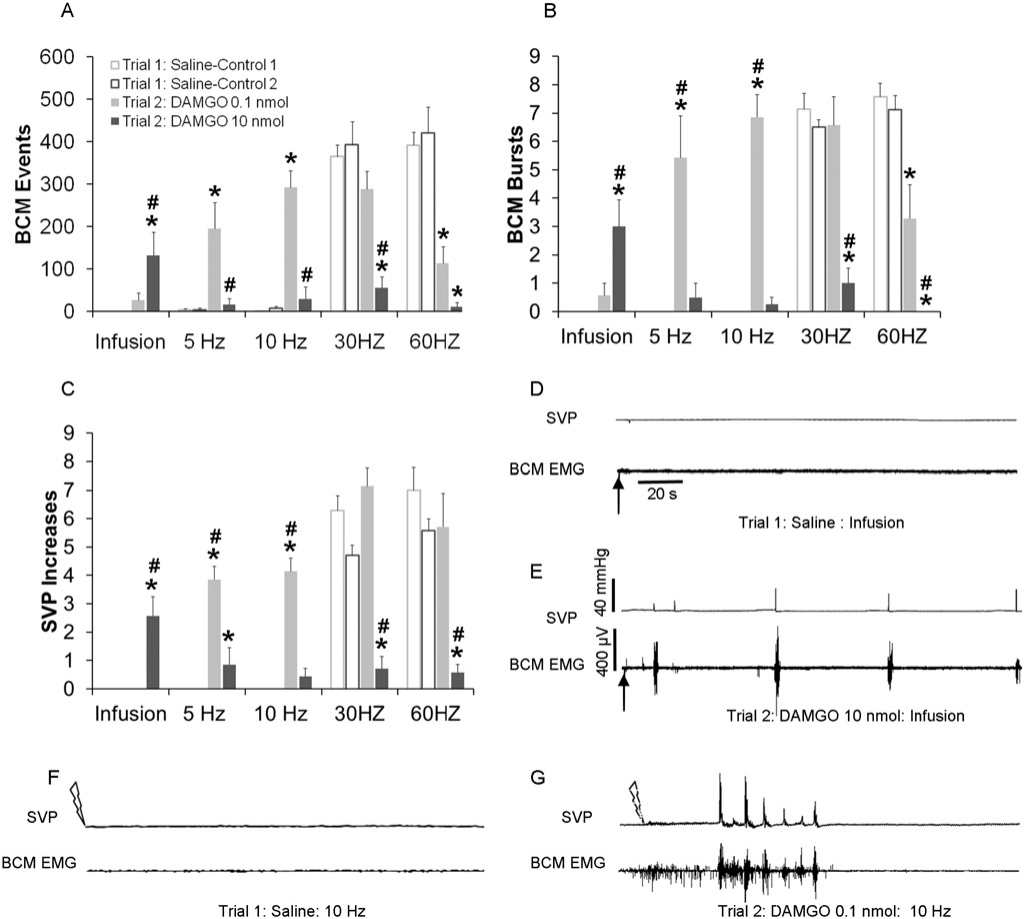
Quantitative analyses of BCM events (A) bursts (B) and SVP increases (C) in response to infusion, 5 Hz, 10 Hz, 30 Hz and 60 Hz DPN stimulation following intrathecal infusions of saline in trial 1 (Saline-Controls 1 and 2; white bars) or one of two doses of DAMGO (0.1 or 10 nmol) in trial 2 (drug trial; filled bars). Trials 1 and 2 were conducted in the same animals, hence Saline-Control 1 are data for animals subsequently receiving DAMGO 0.1 nmol in trial 2 and Saline-Control 2 are data for animals receiving DAMGO 10 nmol in trial 2. Note absence of white bars for infusions, 5, and 10 Hz, as these stimulation frequencies did not trigger BCM events, bursts, nor SVP increases in control conditions. Data are presented as Mean ± SEM. * denotes significant differences from trial 1 within the same treatment group, while # indicates significant differences between treatment groups within the same testing trial. Representative BCM EMG traces (180 seconds duration) following an intrathecal infusion of saline (**D**: control trial) and DAMGO (**E**: 10 nmol: same animal as in D) in the absence of DPN stimulation. EMG and concurrent SVP traces (90 seconds duration) following 10 Hz DPN stimulation (arrow) after an intrathecal infusion of saline (**F**: control trial) and DAMGO (**G**: 0.1 nmol: same animal as in F).

The effects of Mu opioid receptor agonist DAMGO on ejaculatory reflexes was examined in conditions in which such reflexes are not triggered in control conditions; i.e. sub threshold DPN stimulation (5–10 Hz), or in the absence of DPN stimulation (Infusion only). In addition, effects of DAMGO were tested on ejaculatory reflexes triggered by DPN stimulation parameters that reliably trigger such reflexes (30–60 Hz). The mu opioid receptor agonist DAMGO triggered BCM events, bursts and SVP increases in the absence of DPN stimulation (Infusion; Fig. 5). In addition, DAMGO dose-dependently facilitated BCM events, bursts and SVP increases induced by subthreshold levels of DPN stimulation (5–10 Hz; Fig. 5). For the numbers of BCM events, there were main effects of testing trial for all stimulation frequencies (Infusion (0 Hz: (F(1, 29) = 6.400; *P* = 0.025); 5 Hz: (F(1, 29) = 11.407; *P* = 0.005); 10 Hz:(F(1, 29) = 41.312; *P* < 0.001); 30 Hz (F(1, 29) = 42.443; *P* < 0.001); and 60 Hz (F(1, 29) = 73.256; *P* < 0.001); Fig. 5A). Moreover, there were effects of drug dosage (5 Hz: (F(1, 29) = 8.637; *P* = 0.012), 10 Hz:(F(1, 29) = 29.005; *P* < 0.001) and 30 Hz (F(1, 29) = 4.763; *P* = 0.048). DAMGO induced BCM events in the absence of DPN stimulation, but only following the higher dose (10 nmol) and in 100% of males (Infusion: *P* = 0.009 compared to saline in trial 1; p = 0.024 compared to lower dosage in trial 2; Fig. 5A). DAMGO treatment also induced BCM events following subthreshold DPN stimulation; but this effect was only observed with the lower dosage (0.1 nmol) and not the higher dose (10 nmol) (5–10 Hz: *P*< 0.001; compared to saline treatment in trial 1; 5–10 Hz: *P <* 0.001 compared to higher dosage in trial 2; Fig. 5A). In contrast, DAMGO reduced numbers of BCM events following 30 and 60 Hz DPN stimulation frequencies, which reliably triggered BCM activity following saline in trial 1 (Fig. 5A; 30Hz: *P <* 0.001, 10 nmol compared to saline in trial 1 and compared to lower dosage in trial 2; 60 Hz: *P*< 0.001, 10 nmol; *P*< 0.001, 0.1 nmol; compared to saline in trial 1).

The effects of DAMGO on BCM bursts were nearly identical to those described above for BCM events with main effects of testing trial and drug dosage for all stimulation frequencies except 30 Hz (Fig. 5B; Testing trial: Infusion, 0 Hz: (F(1, 29) = 10.726; *P* = 0.006; 5 Hz: (F(1, 29) = 16.136; *P* = 0.001; 10 Hz:(F(1, 29) = 80.755; *P* < 0.001; and 60 Hz (F(1, 29) = 78.692; *P* < 0.001); Drug dosage: and main effects of drug dosages: Infusion: F(1, 29) = 4.960; *P* = 0.044; 5 Hz: (F(1, 29) = 11.152; *P* = 0.005; 10 Hz:(F(1, 29) = 69.792; *P* < 0.001; and 60 Hz (F(1, 29) = 8.074; *P* = 0.014). Post hoc tests showed that DAMGO induced BCM bursts in the absence of DPN stimulation in males treated with the higher dose (10 nmol) but not the lower dose (0.1 nmol; Infusion: *P* = 0.001; compared to saline in trial 1; *P =* 0.001 compared to lower dosage in trial 2, Fig. 5B). DAMGO also facilitated BCM bursts following subthreshold DPN stimulations, but only with the lower (0.1 nmol) and not the higher (10 nmol) dose (5 Hz: *P*< 0.001; 10 Hz *P*< 0.001; compared to saline in trial 1 or higher dose in trial 2, Fig. 5B). DAMGO reduced numbers of BCM bursts following 60 Hz DPN stimulation (lower dose (0.1 nmol): *P <* 0.001 compared to saline in trial 1; higher dose (10 nmol): *P <* 0.001 compared to saline in trial 1 and *P =* 0.014 compared to lower dose in trial 2, Fig. 5B).

Finally, effects of DAMGO on SVP increases mirrored those on BCM activity (Fig. 5C). There were main effects of testing trial (Infusion (0 Hz: (F(1, 27) = 8.168; *P* = 0.014; 5 Hz: (F(1, 27) = 28.106; *P* < 0.001; 10 Hz:(F(1, 27) = 63.157; *P* < 0.001; 30 Hz (F(1, 27) = 6.866; *P* = 0.022; and 60 Hz (F(1, 27) = 16.471; *P* < 0.002) and of drug dosage (Infusion: (F(1, 27) = 8.168; *P* = 0.014; 5 Hz: (F(1, 27) = 5.807; *P* = 0.033; 10 Hz:(F(1, 27) = 31.383; *P* < 0.001; 30 Hz:(F(1, 27) = 18.103; *P* = 0.001; and 60 Hz:(F(1, 27) = 9.874; *P* = 0.008). DAMGO infusions alone, in the absence of DPN stimulation, caused increases in SVP in males treated with the higher dose (10 nmol) but not the lower dose (0.1 nmol) (Infusion: *P*< 0.001; compared to saline in trial 1 or to the lower dose in trial 2, Fig. 5C). Furthermore, DAMGO increased SVP following subthreshold DPN stimulation (5 Hz: *P* < 0.001, 0.1 nmol; *P* = 0.047, 10 nmol; 10 Hz: *P*< 0.001; 0.1 nmol) compared to saline treatment in trial 1. The lower dosage of DAMGO was significantly more effective in causing SVP increases than the higher dosage (5 Hz: *P <* 0.001; 10 Hz: *P <* 0.001). Lastly, the higher dose of DAMGO (10 nmol) decreased SVP increases induced by threshold DPN stimulation (30 Hz (*P*< 0.001) and 60 Hz (*P*< 0.001) compared to saline in trial 1 or to the lower dose in trial 2, Fig. 5C).

During trail 1 (saline control), there were no significant differences in BCM events, bursts, or SVP increases between groups, indicating that the differences observed in trial 2 were due to the effects of DAMGO.

### Delta Opioid Receptor Agonist Deltorphin II Facilitated Ejaculatory Reflexes

**Fig 6:**
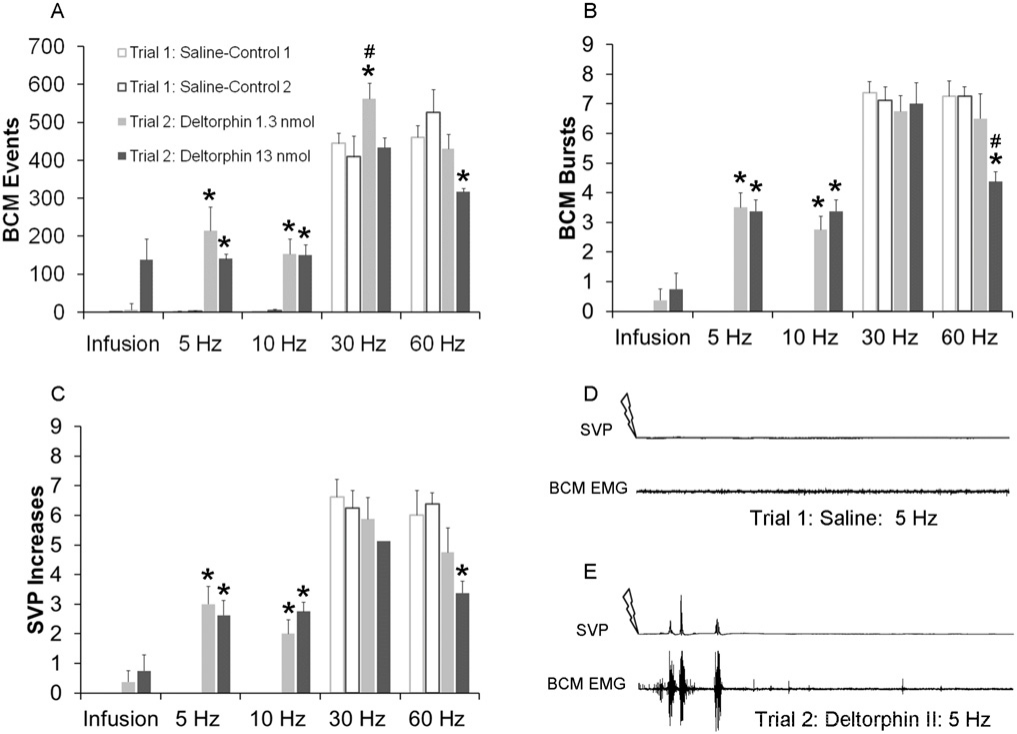
Quantitative analyses of BCM events (A) bursts (B) and SVP increases (C) following infusion, 5 Hz, 10 Hz, 30 Hz and 60 Hz DPN stimulation after intrathecal infusions of saline in trial 1 (Saline-Controls 1 and 2; white bar) or deltorphin II (1.3 or 13 nmol) in trial 2 (drug trial; filled bars). Trials 1 and 2 were conducted in the same animals, hence Saline-Control 1 are data for animals subsequently receiving deltorphin II 1.3 nmol in trial 2 and Saline-Control 2 are data for animals receiving deltorphin II 13nmol in trial 2. Data are presented as Mean ± SEM. Note absence of white bars for infusions, 5, and 10 Hz, as these stimulation frequencies did not trigger BCM events, bursts, nor SVP increases in control conditions.* denotes significant differences from trial 1 (control trial), while # indicates significant differences from lower dosage within the same testing trial. BCM EMG and SVP traces (90 seconds duration) following 5 Hz DPN stimulation (arrow) after an intrathecal infusion of saline (**D**: control trial) and deltorphin II (**E**: same animal as in D).

In contrast to the effects of DAMGO, deltorphin II did not induce BCM activity or SVP increases in the absence of DPN stimulation (Infusion; Fig. 6). However, deltorphin II enhanced the emission and expulsion components of the ejaculatory reflex following subthreshold DPN stimulation (5–10 Hz; Fig. 6). There were main effects of testing trial on BCM events (5 Hz: (F(1, 31) = 33.286; *P* < 0.001; 10 Hz:(F(1, 31) = 56.543; *P* < 0.001; 30 Hz (F(1, 31) = 6.059; *P* = 0.027; 60 Hz (F(1, 31) = 5.710; *P* < 0.031; Fig. 6A), BCM bursts (5 Hz: (F(1, 31) = 121.00; *P* < 0.001; 10 Hz:(F(1, 31) = 108.432; *P* < 0.001; 60 Hz (F(1, 31) = 14.608; *P* < 0.002; Fig. 6B), and SVP increases (5 Hz: (F(1, 31) = 52.417; *P* < 0.001; 10 Hz:(F(1, 31) = 54.000; *P* < 0.001; and 60 Hz (F(1, 31) = 8.590; *P* = 0.011; Fig. 6C), and a significant interaction between testing trial and drug dosage on BCM bursts for 60 Hz (F(1, 31) = 5.020; *P* = 0.042) stimulation. Deltorphin II (1.3 nmol or 13 nmol) significantly increased numbers of BCM events, burst and SVP increases following 5 Hz (events: 1.3 nmol: *P*< 0.001; 13 nmol:*P* = 0.006; bursts: 1.3 nmol: *P*< 0.001; 13 nmol: *P*< 0.001; SVP:1.3 nmol: *P*< 0.001; 13 nmol: *P*< 0.001; Fig. 6A-E) and 10 Hz (events: 1.3 nmol: *P*< 0.001; 13 nmol: *P*< 0.001; bursts: 1.3 nmol: *P*< 0.001; 13 nmol: *P*< 0.001; SVP:1.3 nmol: *P*< 0.001; 13 nmol: *P*< 0.001; Fig. 6C) stimulation frequencies. Deltorphin II did also slightly increase BCM events following 30 Hz, but only after the lower dosage (*P =* 0.012), and without effects on BCM bursts or SVP increases. Finally, the higher dose of deltorphin II (13 nmol) reduced BCM activity and SVP increases following 60 Hz DPN stimulation (events: *P* = 0.011; bursts: *P*< 0.001; SVP increases: *P* = 0.011) compared to saline treatment in trial 1. As noted for each of the experiments presented above, there were again no differences in BCM or SVP parameters between groups during the saline control trial 1.

## Discussion

The results of these experiments support the hypothesis that enkephalin plays a critical role in the control of ejaculation by acting on mu and delta opioid receptors in LSt target areas in the lumbosacral spinal cord. Antagonist studies showed that activation of either mu or delta opioid receptors is critical for the emission and expulsion phases of ejaculation in response to sensory stimulation in male rats. In addition, activation of either mu or delta receptor by agonist administration at lower dosages facilitated both the emission and expulsion phases of ejaculation following sub- threshold sensory stimulation. Finally, stimulation of mu, but not delta, opioid receptors was sufficient to trigger ejaculatory reflexes in the absence of sensory stimulation. Together, these findings demonstrate that endogenous activation of mu or delta opioid receptors plays a facilitative role in the control of ejaculation.

A first main finding of the current study was that intrathecal infusions of either mu or delta opioid antagonists CTOP or TIPP, severely disrupted ejaculatory reflex induced by DPN stimulation, using nerve stimulation parameters that reliable triggered ejaculation under control conditions. Conversely, facilitation of ejaculatory reflexes by infusions of mu or delta receptor agonists DAMGO or deltorphin II, was observed after DPN stimulation at sub-threshold parameters that do not trigger ejaculation in control animals [14–16,25]. These data support our hypothesis that endogenous opioids are acting in LSt target areas to regulate ejaculatory reflexes. This was further confirmed by the finding that antagonist infusions did not disrupt activation of LSt cells, indicating that opioid antagonists acted on neurons downstream from LSt cells, rather than on inputs to LSt cells. The exact locations of the opioid receptors within the spinal ejaculation generator are currently unknown. Hence, a detailed examination of the localization of mu and delta receptors specifically within the LSt target areas is a critical next step. The finding that both mu and delta receptors are involved in mediating ejaculation, indicates that enkephalin may act on both receptors and suggest the possibility of interactions between the opioid receptors during control of ejaculation. Indeed, studies have shown that opioid receptors may form heterodimers [34–36]. Mu and delta opioid receptors demonstrate a high degree of sequence homology and a common opioid receptor binding site within the helical transmembrane core has been proposed to account for the ligand-directed signaling or biased agonism which occurs at opioid receptors [36]. Moreover, there is evidence for cooperation between delta and mu opioid receptors [37], such that activation of one receptor causes allosteric enhancement of ligand binding and activity of the other receptor [38].

Another of the main findings of the current study was that intrathecal infusion of the higher dose of mu opioid receptor agonist DAMGO, but not delta opioid receptor agonist, was sufficient to trigger ejaculatory reflexes in the absence of sensory stimulation in a large proportion of male rats (75%). Mu and delta opioid receptors are inhibitory G-protein coupled receptors [39]; therefore, it is possible that the mechanism of action whereby endogenous opioids trigger or facilitate ejaculatory reflexes is by means of inhibition of inhibitory neurons in LSt target regions. For example, opioids can exert analgesia through inhibition of inhibitory GABA (γ—aminobuteric acid) neurons [40]. Opioids inhibit GABA-mediated synaptic transmission by reducing the likelihood of presynaptic neurotransmitter release [41,42] and this effect is mediated by voltage-dependent potassium conductance [42]. Mu but not delta opioid receptors are specifically coupled to this potassium conductance [43] and the subsequent inhibition of GABAergic synaptic transmission; therefore we speculate that this may explain that intrathecal infusions of DAMGO, a selective MOR agonist, but not deltorphin II, a selective DOR agonist, triggered ejaculatory reflexes in the absence of DPN stimulation. However, localization of presynaptic MOR on LSt axons has not been confirmed and further studies identifying the exact pre- or postsynaptic localization of these receptors are critical to elucidate the mechanisms by which opioids influence ejaculatory function.

In apparent contrast to the inhibitory effects of opioid receptor antagonists and the facilitative effects of the lower dosages of the opioid receptor agonists, infusions of higher dosages receptor agonists for either the mu or delta opioid receptor disrupted the ability of DPN stimulation to trigger ejaculation. This finding is in conformity with previous studies which showed that systemic administration of morphine exerts inhibitory effects on ejaculatory behavior [44] and reflexes in male rats [26]. The effects of systemic morphine have been proposed to be partially mediated via actions on peripheral tissues [27]. The current findings demonstrate that the inhibitory effects of intrathecal administration of high doses of opioid receptor agonists are mediated via central actions within the lumbosacral spinal cord. Noteworthy, our previous studies showed similar inhibitory effects of higher doses of GRP agonists on ejaculatory reflexes, while lower doses of GRP agonists triggered or facilitated ejaculation. Therefore, it is possible that high doses of GRP or opioid receptor ligands cause mechanisms of desensitization of the G-protein coupled receptors at the LST target sites [45]; thereby preventing the ability of DPN stimulation to trigger further action of endogenous ligands on receptor activation. It is also possible that infusion of the higher dose of mu and delta receptor agonists prevented DPN stimulation-induced ejaculatory reflexes by acting on the processing of the sensory inputs relayed via the DPN. The DPN is comprised of A δ and C-fibers [46] but the specific contributions of A δ and C-fibers to ejaculation have not been investigated. Intrathecal infusions of opioid agonists inhibit A δ and C-fibers [47] raising the possibility that the disruption of ejaculatory reflexes to threshold sensory stimulation after the higher dose but not the lower dose of opioid agonists is mediated by inhibition of A δ and C-fibers in the dorsal horn of the lumbosacral spinal cord. In addition, the cell bodies of A δ and C-fibers in the dorsal root ganglia express mu and delta opioid receptors [48], indicating that presynaptic inhibition of DPN synaptic transmission may have contributed to disruption of ejaculation following intrathecal infusions of higher dosages of opioid receptor agonists. Finally, the primary excitatory neurotransmitter released from A δ and C-fibers is glutamate [39] which activates NMDA receptors in LSt cells to trigger ejaculatory reflexes in male rats [15]. Opioids in turn, can inhibit the release of glutamate [39] thereby potentially blocking the activation of LSt cells in the lumbosacral spinal cord. This possibility was not tested in the current study and requires further investigation.

In conclusion, these data support the hypothesis that activation of mu and delta opioid receptors in the lumbosacral spinal cord is required for sensory stimulation-induced emission and expulsion in anesthetized and spinalized male rats. The endogenous opioid ligand enkephalin may act through the activation of both mu and delta receptors in the outputs of the LSt cells. However, precise localization of mu and delta opioid receptor expression in the spinal ejaculation generator is currently unknown and needs to be examined in detail in future studies. Furthermore, the roles of other neuropeptides expressed in the LSt cells and axons, including galanin and cholecystokinin, in the control of ejaculation remain an open question. Finally, these data indicate that mu and delta opioid antagonists may be useful for the treatment of ejaculatory dysfunction, specifically to delay the onset of ejaculation in men afflicted with premature ejaculation.
